# NMR side-chain assignments of the Crimean–Congo hemorrhagic fever virus glycoprotein n cytosolic domain

**DOI:** 10.5194/mr-5-95-2024

**Published:** 2024-07-08

**Authors:** Louis Brigandat, Maëlys Laux, Caroline Marteau, Laura Cole, Anja Böckmann, Lauriane Lecoq, Marie-Laure Fogeron, Morgane Callon

**Affiliations:** 1 Molecular Microbiology and Structural Biochemistry (MMSB), UMR5086 CNRS, University of Lyon, 7, passage du Vercors, 69367 Lyon, CEDEX 07, France

## Abstract

We assigned the side-chain resonances of the Crimean–Congo hemorrhagic fever virus (CCHFV) glycoprotein n (Gn) cytosolic domain that is 69 amino acids long to complete the backbone resonances previously published by Estrada et al. (2011). The process was facilitated by three factors. First, sample preparation using cell-free protein synthesis (CFPS) was completed in less than 2 d and allowed for correct zinc finger formation by adding zinc ions to the reaction. Second, access to NMR platforms with standardized pulse sequences allowed for data acquisition in 18 d. Third, data analysis using the online platform NMRtist allowed sequential resonance assignments to be made in a day, and assignments were verified and finalized in less than a week. Our work thus provides an example of how NMR assignments, including side chains, of small and well-behaved proteins can be approached in a rapid routine, at protein concentrations of 150 µ M.

## 1 Introduction

The Bunyavirales order is a large group of serologically related, often tripartite, negative-sense RNA viruses. Crimean–Congo hemorrhagic fever virus (CCHFV), a member of the order, is considered one of the most deadly viruses, with a mortality rate of up to 40 % (Ye et al., 2022; Hawman and Feldmann, 2023). CCHFV is endemic in most parts of Africa, in the Balkans, in the Middle East and in Asia (Shahhosseini et al., 2021). It poses a serious threat to both human and animal health, and it is currently spreading to non-endemic areas as their vectors expand their range with global warming. Indeed, the *Hyalomma marginatum* ticks that transmit CCHFV are already present in Europe (Bonnet et al., 2022), and the virus has caused human fatalities in Spain (Lorenzo Juanes et al., 2023); it also has been recently (2023) detected for the first time in southern France (Bernard et al., 2024).

The three genomic segments of most Bunyavirales can be divided into the small (S), the medium (M) and the large (L) segments. Bunyavirales often share a similar protein composition, consisting of the nucleocapsid protein (NP), which forms the ribonucleoprotein (RNP) complex with the viral genomes; the two envelope proteins glycoprotein n (Gn) and glycoprotein c (Gc); and the RNA-dependent RNA polymerase (RdRp). Structural information is abundant for NP (e.g., Sun et al., 2018; Arragain et al., 2019) and also for RdRp (e.g., Arragain et al., 2019, 2020, 2022). The Gn and Gc envelope glycoproteins are inserted into the endoplasmic reticulum (ER) during translation and eventually localize to the surface of the viral particle, forming the trimeric spikes that characterize mature particles. Gn and Gc have a large ectodomain, a transmembrane anchor and a small cytosolic (cyto) domain (Hulswit et al., 2021). In particular, the Gc ectodomain has been extensively studied (Zhu et al., 2017; Guardado-Calvo and Rey, 2017; Bignon et al., 2019; Mishra et al., 2022). In contrast, its transmembrane and cytosolic domains (Gc ${}^{\mathrm{TMcyto}}$ ) have not yet been structurally characterized. For some viruses, information is available for the Gn ectodomain (Hastie et al., 2017; Hulswit et al., 2021). The Gn ectodomain interacts with the one of Gc and undergoes extensive structural changes during maturation (Halldorsson et al., 2018). Several structures exist for the isolated cytosolic domain of Gn (Gn ${}^{\mathrm{cyto}}$ ), typically from hantavirus, CCHFV and Junín virus (Estrada et al., 2009; Estrada and De Guzman, 2011). This domain contains a double ββα zinc finger (ZF) motif that could play a matrix protein role in viral particle assembly (Strandin et al., 2013), as does the Lassa virus Z protein, which alone is sufficient for the release of virus-like particles (Strecker et al., 2003).

The sequential NMR resonance assignments of the (69 amino acid long) CCHFV Gn ${}^{\mathrm{cyto}}$ have been reported in the literature, and they were deposited in the Biological Magnetic Resonance Bank (BMRB) under accession number 17383 (Estrada and De Guzman, 2011). Amide proton and ${}^{13}$ C/ ${}^{15}$ N backbone resonances are presented there, alongside C β assignments. However, other ${}^{13}$ C side-chain resonances as well as protons remained unassigned. Because of our interest in eventually comparing the isolated domain with the membrane-bound form and its likely multimeric states, which we will address using proton-detected magic-angle-spinning NMR, we now report here the ${}^{1}$ H and ${}^{13}$ C side-chain resonance assignments, as well as the cell-free sample preparation procedures we used to efficiently prepare the protein domain.

## 2 Materials and methods

### 2.1 Wheat-germ cell-free protein synthesis (WG-CFPS)

All samples were produced using WG-CFPS. The sequence corresponding to the cytosolic domain of Gn (residues 733–799, strain IbAr10200) was cloned into the pEU-E01-MCS vector (CellFree Sciences, Japan) with a Strep-tag fused at its C terminus. The plasmid was then amplified in DH5 α cells and purified using a NucleoBond Xtra Maxi kit (Macherey-Nagel, France). Further purification was performed by phenol–chloroform extraction to achieve the required level of plasmid purity. The mRNA transcription was performed using 100 ng µ L ${}^{-1}$ plasmid, 2.5 mM NTP mix (Promega, France), 1 U µ L ${}^{-1}$ RNase inhibitor (U represents units) (CellFree Sciences, Japan) and 1 U µ L ${}^{-1}$ SP6 RNA polymerase (CellFree Sciences, Japan) in transcription buffer containing 80 mM Hepes KOH (pH 7.6), 16 mM magnesium acetate, 10 mM DTT and 2 mM spermidine (CellFree Sciences, Japan). The solution was incubated at 37 °C for 6 h, and the mRNA produced was used directly for translation. Cell-free synthesis was performed with uncoupled transcription and translation. All steps followed the protocol of Takai and colleagues (Fogeron et al., 2017; Takai et al., 2010). Translation reactions were performed in a six-well plate (6 mL per well, total volume of 36 mL) using the bilayer method for 16 h at 22 °C. Uniform ${}^{2}$ H– ${}^{13}$ C– ${}^{15}$ N or ${}^{13}$ C– ${}^{15}$ N-labeled amino acids (Cambridge Isotope Laboratories) were used for sample preparations. The sample was purified by affinity chromatography using a 1 mL *Strep*-Tactin© Superflow© gravity flow column (IBA Lifesciences). The protein was eluted in 50 mM phosphate buffer (pH 6.5), 50 mM NaCl and 2.5 mM desthiobiotin, and it was concentrated using a 3 kDa Amicon Ultra filter to 109 µ M ( ${}^{2}$ H– ${}^{13}$ C– ${}^{15}$ N sample) and 150 µ M ( ${}^{13}$ C– ${}^{15}$ N sample) as measured using a NanoDrop. Then, 1× EDTA-free protease inhibitor (cOmplete, EDTA-free protease inhibitor cocktail, Roche) was added to the final sample to avoid protein degradation. Zinc sulfate was added to each buffer in the manufacturing process at a concentration of 100 µ M.

### 2.2 NMR spectroscopy

NMR spectra were acquired at 298 K on Bruker Avance III 600 MHz (3D spectra for backbone assignments) and 950 MHz (3D spectra for side-chain assignments) spectrometers both equipped with cryoprobes. Backbone ${}^{1}$ H, ${}^{15}$ N and ${}^{13}$ C resonance assignments were performed using standard NMR experiments set up via the NMRlib tool (Favier and Brutscher, 2019) in TopSpin 4.0.8 (Bruker Topspin). Specifically, ${}^{15}$ N HSQC, HNCA, HNCO, HNcoCA and HNcoCACB were recorded for backbone assignment, and ${}^{13}$ C HSQC, HccoNH-TOCSY, hCCH-TOCSY and HCcH-TOCSY were recorded for side-chain assignment (a ${}^{15}$ N HSQC was recorded equally on the protonated sample to transfer ${}^{1}$ H– ${}^{15}$ N chemical shifts to the protonated protein and to validate the saturation of the zinc binding sites) (see Table S1 in the Supplement). DSS (sodium trimethylsilylpropanesulfonate) was used for ${}^{1}$ H chemical shift referencing. All spectra were processed using TopSpin 4.0.8 (Bruker Biospin), and spectral analysis and backbone assignment were performed using CcpNmr v3 (Vranken et al., 2005; Stevens et al., 2011; Skinner et al., 2016). Side-chain assignment was performed using NMRtist (https://nmrtist.org/, last access: 26 July 2023) and manually validated using CcpNmr v3.

**Figure 1:**
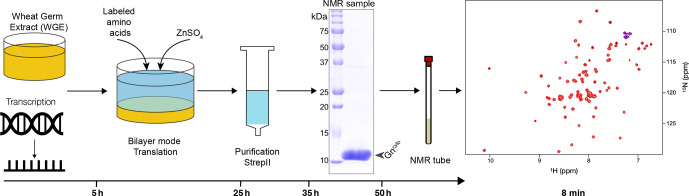
Workflow for sample preparation. Wheat-germ extract and mRNA encoding the protein are used in a bilayer cell-free reaction. Addition of protonated or deuterated ${}^{13}$ C/ ${}^{15}$ N-labeled amino acids allowed for the synthesis of labeled protein. Zinc sulfate was added to the reaction to ensure correct zinc finger formation. After a single-step purification using the C-terminal Strep-II tag with concomitant buffer exchange, the protein showed high purity and was filled into the NMR tube. The entire sample preparation took approximately 2 d. The ${}^{15}$ N-HSQC spectrum was acquired in 8 min.

**Figure 2:**
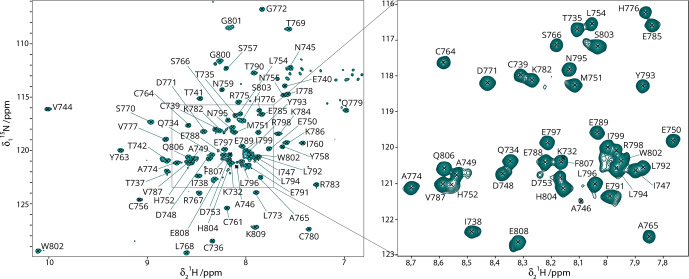
HN backbone assignments. ${}^{15}$ N-HSQC spectrum of ${}^{13}$ C– ${}^{15}$ N Gn ${}^{\mathrm{cyto}}$ with the assigned residues indicated. Assignments could mostly be transferred from the BMRB entry 17383 (Estrada and De Guzman, 2011). All resonance assignments of backbone amides were verified by 3D NMR spectra recorded for the ${}^{2}$ H– ${}^{13}$ C– ${}^{15}$ N-labeled sample.

## 3 Results

### 3.1 Efficient NMR sample preparation using wheat-germ cell-free protein synthesis

Wheat-germ cell-free protein synthesis (CFPS) was used to prepare all NMR samples. This allowed for rapid and efficient preparation of the protein domain and provided sufficient amounts for NMR. Since it has been shown that supplementation with zinc ions significantly increases the solubility and stability of zinc-binding proteins (Matsuda et al., 2006; Jirasko et al., 2020), we investigated whether the addition of ZnSO ${}_{4}$ stabilizes the fold of the Gn zinc finger (ZF) domain by using circular dichroism (CD) and the addition of EDTA to determine under which conditions the folded protein is obtained (Fig. S1 in the Supplement). The CD spectra clearly show that the zinc finger formation is EDTA-dependent and reversible, and that the fold appears more stable when ZnSO ${}_{4}$ is added. This shows that folded Gn ${}^{\mathrm{cyto}}$ can be obtained in CFPS by simply adding ZnSO ${}_{4}$ to the reaction. A zinc sulfate concentration of 100 µ M was shown to be sufficient for complete saturation of the zinc binding sites (see Fig. S2). Fig. 1 summarizes the sample preparation workflow, the pure protein preparation used to record the spectra and the HSQC fingerprint spectrum obtained.

### 3.2 NMR backbone assignments

The backbone assignments for the H ${}^{N}$ , N ${}^{H}$ , C α , Cß, and H α of Gn ${}^{\mathrm{cyto}}$ have been reported in the literature (Estrada and De Guzman, 2011). In a first step, we analyzed the protein sample to confirm that the zinc finger fold was correctly obtained in the cell-free sample by comparing the NMR chemical shifts obtained here to previous data. To do this, we prepared a deuterated sample and recorded fingerprint and backbone correlation spectra, including ${}^{15}$ N-HSQC, HNCA, HNCO, HNcoCA and HNcoCACB. The HNCO was used to add the carbonyl assignments not previously determined. The ${}^{15}$ N-HSQC spectra looked quite different at first sight (Fig. S3), but this was mainly due to the use of different tags and also slightly different viral strains (IbAr10200 in this study vs. SPU103/87 in Estrada and De Guzman, 2011; Fig. S3). The remaining differences might be due to the small variation in pH (7 versus 6.45). The chemical shift perturbations (CSPs) are shown in Fig. S4. Large CSPs between previous assignments and our sample were observed for residues K732, T737, A746, H752, K786 and I799. While the differences for the C-terminal I799 are likely due to the use of a different tag, the differences for the other residues remain unexplained. Most of the other CSPs remain below 0.2 ppm.

**Figure 3:**
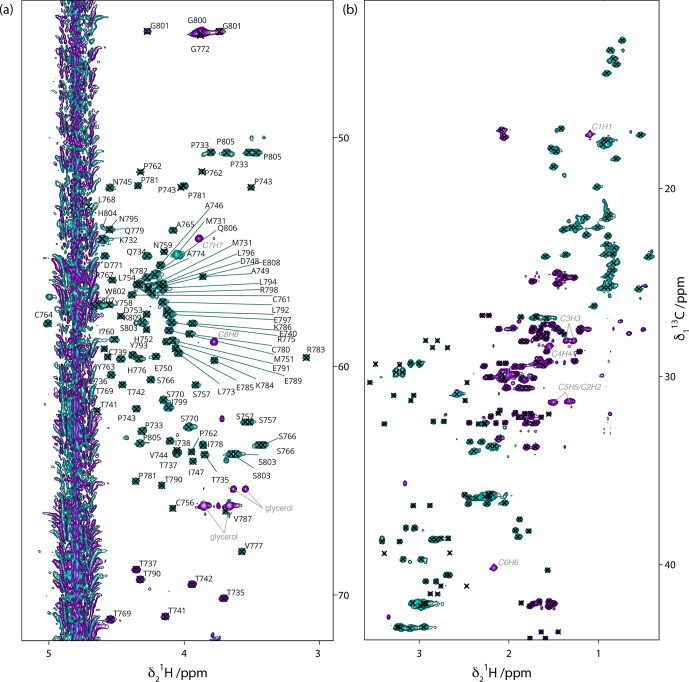
${}^{13}$ C-HSQC spectrum of ${}^{13}$ C– ${}^{15}$ N Gn ${}^{\mathrm{cyto}}$ . **(a)** C α /H α region with assignments shown on the spectrum. **(b)** Side-chain region, with crosses indicating assigned peaks. For assignments, see BMRB submission. Few impurities are observed, which correspond to desthiobiotin (labeled in italic grey) and glycerol (labeled in grey).

### 3.3 NMR side-chain assignments supported by NMRtist

Since we are ultimately interested in further analysis of the domain in its membrane-bound form using solid-state NMR and in comparing the free and membrane-bound states of the domain, we set out to determine its chemical shifts on our construct, including the side-chain resonances missing in the previous work. To this end, we used NMRtist, an artificial intelligence-based program that allows for fully automated NMR spectrum assignment (and protein structure determination; Klukowski et al., 2022). NMRtist requires as input the amino acid sequence of the protein of interest and peak lists of 2D and 3D NMR spectra. It is also possible to provide a partial assignment of the protein resonances. As input, we thus prepared peak lists of ${}^{15}$ N-HSQC and 3D HNCA, HNcoCA, HNcoCACB, HCcH, and hCCH spectra.

Automated resonance assignments were tested with and without providing the software with protein backbone assignments as input. Without providing any previous assignments, the software estimates the accuracy of chemical shift assignments at 81 %. This value increases to 89 % when backbone chemical shifts are given as input. The assignment statistic of the latter NMRtist run is shown in Fig. S5.

Peak assignments were manually verified using the Peak Predict module of the CCPN v3 software and by comparing the predicted peaks with the actual spectra, particularly the ${}^{13}$ C-HSQC spectrum. Some residues required small manual shifting. Also, assignments of peaks from impurities such as desthiobiotin and glycerol were added.

We identified several causes that led to (minor) problems in NMRtist assignments. Firstly, the fact that 3D backbone and side-chain spectra were not recorded on the same sample but on ${}^{2}$ H– ${}^{13}$ C– ${}^{15}$ N- and ${}^{13}$ C– ${}^{15}$ N-labeled proteins, respectively, led to some inaccuracies. While the ${}^{15}$ N-HSQC assignments were still very similar and many assignments could be transferred, slight differences due to the difference in labeling induced small errors. An additional error source was the relatively poor resolution of the HNcoCACB, which induced a greater uncertainty in the C α and C β chemical shifts in this spectrum. Most problems were confined to regions where peak overlap occurred. Despite the few errors in the assignment, the use of NMRtist saved a lot of time, as all easy or isolated spin systems were quickly assigned with high confidence. Furthermore, the addition of the manual backbone assignments in NMRtist improved the estimated chemical shift assignment accuracy from 81 % to 90 %. TALOS-N predictions show that the assignments comply with the published NMR structure (PDB 2L7X) (Estrada and De Guzman, 2011) (Fig. S6).

In the end, most of the side-chain carbon and proton assignments could be achieved, as shown in the extracts of the ${}^{13}$ C-HSQC spectrum in Fig. 3, where the assignments for the H α /C α are given, and all assigned side-chain peaks are marked with a cross. The data have been deposited at the BMRB under accession number 52372.

## 4 Discussion and conclusion

We have shown here that the production and resonance assignment of small protein domains can be done in a highly efficient manner by combining cell-free protein synthesis, NMR spectroscopy on high-field platforms and automated resonance assignment. The use of a high magnetic field not only provided the high resolution which facilitated the assignment of side-chain protons, but also provided the sensitivity to study the below 150 µ M samples supplied by CFPS. While the sample preparation took about 2 d, the acquisition of the side-chain assignment spectra took 2–3 weeks and the analysis took 1–2 weeks to verify the automated assignments. The backbone assignments were not very time-consuming since they had been previously established (Estrada and De Guzman, 2011), but they required verification, which took several days when done manually but was not necessary for automated assignments, which were similarly successful in the absence of this prior knowledge.

The protein considered here is rather small, with 69 amino acids plus a 10 amino acid tag, and represents a subdomain of a larger, membrane-bound protein. While solid-state NMR can address the membrane-bound forms, solution-state NMR spectra of the isolated domains have the potential to accelerate a future analysis of the solid-state NMR 2D hCH and further 3D spectra, such as hCCH. The knowledge gained here will also be used to analyze the differences between the isolated and membrane-bound domains as highlighted by CSPs.

The number of situations where small domains can provide partial information on membrane-bound proteins is high in the study of viral proteins, as many viral envelope proteins carry rather small ectodomains or cytosolic domains that are easily accessible to solution NMR. Together with AlphaFold2 predictions, sequential NMR resonance assignments can even obviate the need for structure calculations if the models are of high quality, since the secondary chemical shifts from sequential assignments provide immediate information on the secondary structure. The advantage of cell-free protein synthesis is that both membrane-bound and isolated domains can be produced with similar efficiency using the same approach.

## Supplement

10.5194/mr-5-95-2024-supplement The supplement related to this article is available online at: https://doi.org/10.5194/mr-5-95-2024-supplement.

## Supplement

10.5194/mr-5-95-2024-supplement
10.5194/mr-5-95-2024-supplement The supplement related to this article is available online at: https://doi.org/10.5194/mr-5-95-2024-supplement.

## Data Availability

Chemical shifts are submitted to the BMRB with the accession number 52372 (Laux and Böckmann, 2024). NMR spectra have been deposited to 10.5281/zenodo.10938432 (Lecoq et al., 2024).

## Appendix

The supplement related to this article is available online at: https://doi.org/10.5194/mr-5-95-2024-supplement.

## References

[bib1.bib1] Arragain B, Reguera J, Desfosses A, Gutsche I, Schoehn G, Malet H (2019). High resolution cryo-EM structure of the helical RNA-bound Hantaan virus nucleocapsid reveals its assembly mechanisms. eLife.

[bib1.bib2] Arragain B, Effantin G, Gerlach P, Reguera J, Schoehn G, Cusack S, Malet H (2020). Pre-initiation and elongation structures of full-length La Crosse virus polymerase reveal functionally important conformational changes. Nat Commun.

[bib1.bib3] Arragain B, Durieux Trouilleton Q, Baudin F, Provaznik J, Azevedo N, Cusack S, Schoehn G, Malet H (2022). Structural snapshots of La Crosse virus polymerase reveal the mechanisms underlying Peribunyaviridae replication and transcription. Nat Commun.

[bib1.bib4] Bernard C, Joly Kukla C, Rakotoarivony I, Duhayon M, Stachurski F, Huber K, Giupponi C, Zortman I, Holzmuller P, Pollet T, Jeanneau M, Mercey A, Vachiery N, Lefrançois T, Garros C, Michaud V, Comtet L, Despois L, Pourquier P, Picard C, Journeaux A, Thomas D, Godard S, Moissonnier E, Mely S, Sega M, Pannetier D, Baize S, Vial L (2024). Detection of Crimean–Congo haemorrhagic fever virus in Hyalomma marginatum ticks, southern France, May 2022 and April 2023. Eurosurveillance.

[bib1.bib5] Bignon EA, Albornoz A, Guardado-Calvo P, Rey FA, Tischler ND (2019). Molecular organization and dynamics of the fusion protein Gc at the hantavirus surface. eLife.

[bib1.bib6] Bonnet SI, Vourc'h G, Raffetin A, Falchi A, Figoni J, Fite J, Hoch T, Moutailler S, Quillery E (2022). The control of Hyalomma ticks, vectors of the Crimean–Congo hemorrhagic fever virus: Where are we now and where are we going?. PLoS Negl Trop D.

[bib1.bib7] Estrada DF, De Guzman RN (2011). Structural Characterization of the Crimean-Congo Hemorrhagic Fever Virus Gn Tail Provides Insight into Virus Assembly. J Biol Chem.

[bib1.bib8] Estrada DF, Boudreaux DM, Zhong D, St. Jeor SC, De Guzman RN (2009). The Hantavirus Glycoprotein G1 Tail Contains Dual CCHC-type Classical Zinc Fingers. J Biol Chem.

[bib1.bib9] Favier A, Brutscher B (2019). NMRlib: user-friendly pulse sequence tools for Bruker NMR spectrometers. J Biomol NMR.

[bib1.bib10] Fogeron M-L, Badillo A, Penin F, Böckmann A, Lacapere J-J (2017). Membrane Protein Structure and Function Characterization, vol 1635.

[bib1.bib11] Guardado-Calvo P, Rey FA (2017). Advances in Virus Research, vol 98.

[bib1.bib12] Halldorsson S, Li S, Li M, Harlos K, Bowden TA, Huiskonen JT (2018). Shielding and activation of a viral membrane fusion protein. Nat Commun.

[bib1.bib13] Hastie KM, Zandonatti MA, Kleinfelter LM, Heinrich ML, Rowland MM, Chandran K, Branco LM, Robinson JE, Garry RF, Saphire EO (2017). Structural basis for antibody-mediated neutralization of Lassa virus. Science.

[bib1.bib14] Hawman DW, Feldmann H (2023). Crimean–Congo haemorrhagic fever virus. Nat Rev Microbiol.

[bib1.bib15] Hulswit RJG, Paesen GC, Bowden TA, Shi X (2021). Recent Advances in Bunyavirus Glycoprotein Research: Precursor Processing, Receptor Binding and Structure. Viruses.

[bib1.bib16] Jirasko V, Lends A, Lakomek N-A, Fogeron M-L, Weber M, Malär A, Penzel S, Bartenschlager R, Meier BH, Böckmann A (2020). Dimer organization of membrane-associated NS5A of hepatitis C virus as determined by highly sensitive 1H-detected solid-state NMR. Angewandte Chemie International Edition.

[bib1.bib17] Klukowski P, Riek R, Güntert P (2022). Rapid protein assignments and structures from raw NMR spectra with the deep learning technique ARTINA. Nat Commun.

[bib1.bib18] Laux M, Böckmann A (2024). https://bmrb.io/data_library/summary/index.php?bmrbId=52372.

[bib1.bib19] Lecoq L, Brigandat L, Callon M (2024). Zenodo [data set].

[bib1.bib20] Lorenzo Juanes HM, Carbonell C, Sendra BF, López-Bernus A, Bahamonde A, Orfao A, Lista CV, Ledesma MS, Negredo AI, Rodríguez-Alonso B, Bua BR, Sánchez-Seco MP, Muñoz Bellido JL, Muro A, Belhassen-García M (2023). Crimean-Congo Hemorrhagic Fever, Spain, 2013–2021. Emerg Infect Dis.

[bib1.bib21] Matsuda T, Kigawa T, Koshiba S, Inoue M, Aoki M, Yamasaki K, Seki M, Shinozaki K, Yokoyama S (2006). Cell-free synthesis of zinc-binding proteins. Journal of Structural and Functional Genomics.

[bib1.bib22] Mishra AK, Hellert J, Freitas N, Guardado-Calvo P, Haouz A, Fels JM, Maurer DP, Abelson DM, Bornholdt ZA, Walker LM, Chandran K, Cosset F-L, McLellan JS, Rey FA (2022). Structural basis of synergistic neutralization of Crimean-Congo hemorrhagic fever virus by human antibodies. Science.

[bib1.bib23] Shahhosseini N, Wong G, Babuadze G, Camp JV, Ergonul O, Kobinger GP, Chinikar S, Nowotny N (2021). Crimean-Congo Hemorrhagic Fever Virus in Asia, Africa and Europe. Microorganisms.

[bib1.bib24] Skinner SP, Fogh RH, Boucher W, Ragan TJ, Mureddu LG, Vuister GW (2016). CcpNmr AnalysisAssign: a flexible platform for integrated NMR analysis. J Biomol NMR.

[bib1.bib25] Stevens TJ, Fogh RH, Boucher W, Higman VA, Eisenmenger F, Bardiaux B, Rossum B-J, Oschkinat H, Laue ED (2011). A software framework for analysing solid-state MAS NMR data. J Biomol NMR.

[bib1.bib26] Strandin T, Hepojoki J, Vaheri A (2013). Cytoplasmic tails of bunyavirus Gn glycoproteins–Could they act as matrix protein surrogates?. Virology.

[bib1.bib27] Strecker T, Eichler R, Meulen Jter, Weissenhorn W, Dieter Klenk H, Garten W, Lenz O (2003). Lassa Virus Z Protein Is a Matrix Protein Sufficient for the Release of Virus-Like Particles. J Virol.

[bib1.bib28] Sun Y, Li J, Gao GF, Tien P, Liu W (2018). Bunyavirales ribonucleoproteins: the viral replication and transcription machinery. Crit Rev Microbiol.

[bib1.bib29] Takai K, Sawasaki T, Endo Y (2010). Practical cell-free protein synthesis system using purified wheat embryos. Nat Protoc.

[bib1.bib30] Vranken WF, Boucher W, Stevens TJ, Fogh RH, Pajon A, Llinas M, Ulrich EL, Markley JL, Ionides J, Laue ED (2005). The CCPN data model for NMR spectroscopy: development of a software pipeline. Proteins: Structure, Function, and Bioinformatics.

[bib1.bib31] Ye W, Ye C, Hu Y, Dong Y, Lei Y, Zhang F (2022). The structure of Crimean-Congo hemorrhagic fever virus Gc is revealed; many more still need an answer. Virol Sin.

[bib1.bib32] Zhu Y, Wu Y, Chai Y, Qi J, Peng R, Feng W-H, Gao GF (2017). The Postfusion Structure of the Heartland Virus Gc Glycoprotein Supports Taxonomic Separation of the Bunyaviral Families Phenuiviridae and Hantaviridae. J Virol.

