# Supplementary material for: NMR side-chain assignments of the Crimean–Congo hemorrhagic fever virus glycoprotein n cytosolic domain
Source: Magn Reson (Gott). 2024 Jul 8;5(2):95–101. doi: 10.5194/mr-5-95-2024 (PMC12163917; doi:10.5194/mr-5-95-2024)
Supplement: The supplement related to this article is available online at: https://doi.org/10.5194/mr-5-95-2024-supplement. [file mr-5-95-2024-supplement.pdf]

Supplement of Magn. Reson., 5, 95–101, 2024
https://doi.org/10.5194/mr-5-95-2024-supplement
© Author(s) 2024. CC BY 4.0 License.

Open Access
MAGNETIC RESONANCE

Supplement of

NMR side-chain assignments of the Crimean–Congo hemorrhagic fever virus glycoprotein n cytosolic domain

Louis Brigandat et al.

Correspondence to: Morgane Callon (morgane.callon@ibcp.fr)

The copyright of individual parts of the supplement might differ from the article licence.

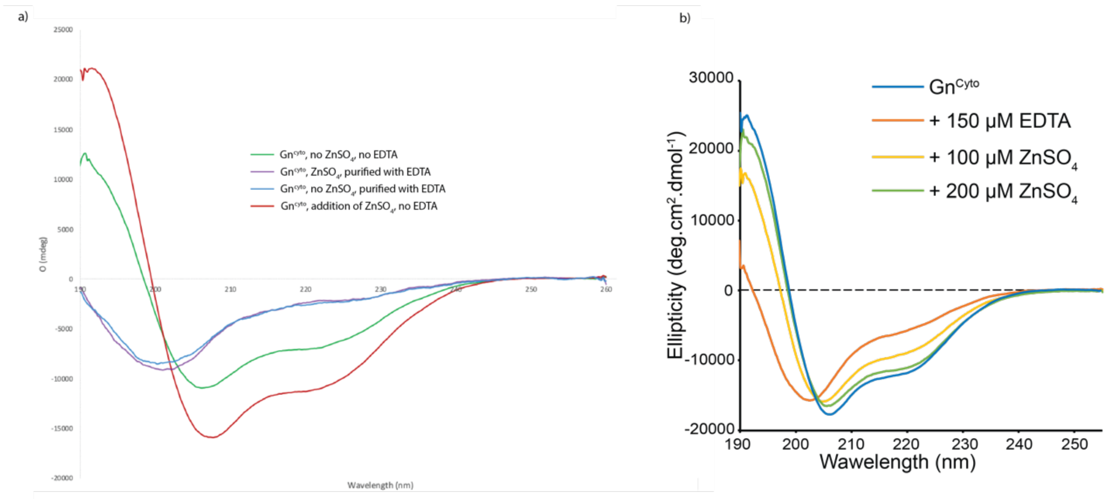

Figure S1: Circular dichroism (CD) spectra of Gn^{cyto} showing **a)** that addition of EDTA causes loss of secondary structure content due to Zn^{2+} depletion, both when synthesized in the presence (purple line) or in the absence (blue line) of $ZnSO_4$. When the protein is synthesized without addition of $ZnSO_4$, secondary structure is observed (green curve), likely by use of ions present in the extract, but the features are less pronounced than when $ZnSO_4$ is added to the cell-free reaction (red curve). **b)** The CD spectrum of Gn^{cyto} recorded on Gn^{cyto} (blue) produced in presence of 100 µM $ZnSO_4$ shows that the secondary structure, which is lost upon addition of 150 µM EDTA (red curve), can be recovered by addition of $ZnSO_4$ (yellow and green curves) to the protein sample after the extraction of Zn^{2+} by EDTA.

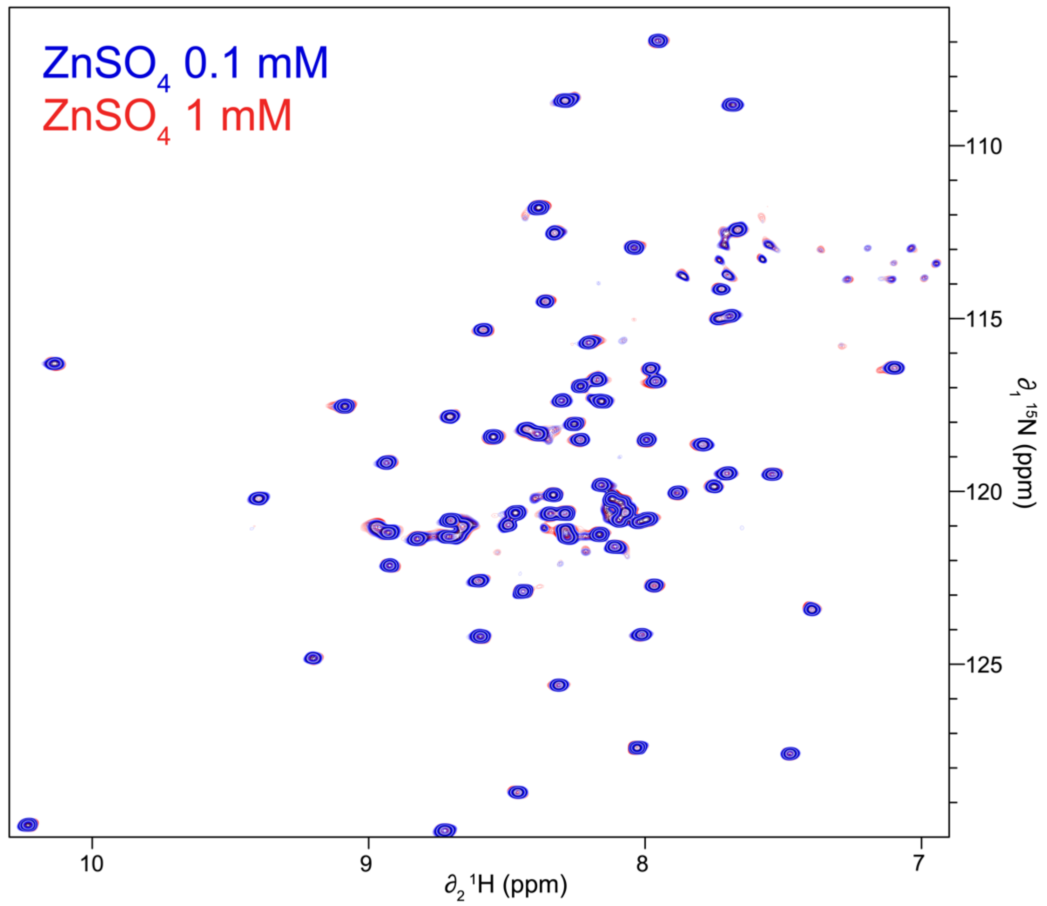

Figure S2: Comparison between the ^{15}N HSQC spectra of ^{13}C-^{15}N Gn^{cyto} at a concentration of 60 µM and a $ZnSO_4$ concentrations of 100 µM (blue) and 1 mM (red). The spectra overlap well, indicating that the Zinc binding sites are already saturated at a $ZnSO_4$ concentration of 100 µM.

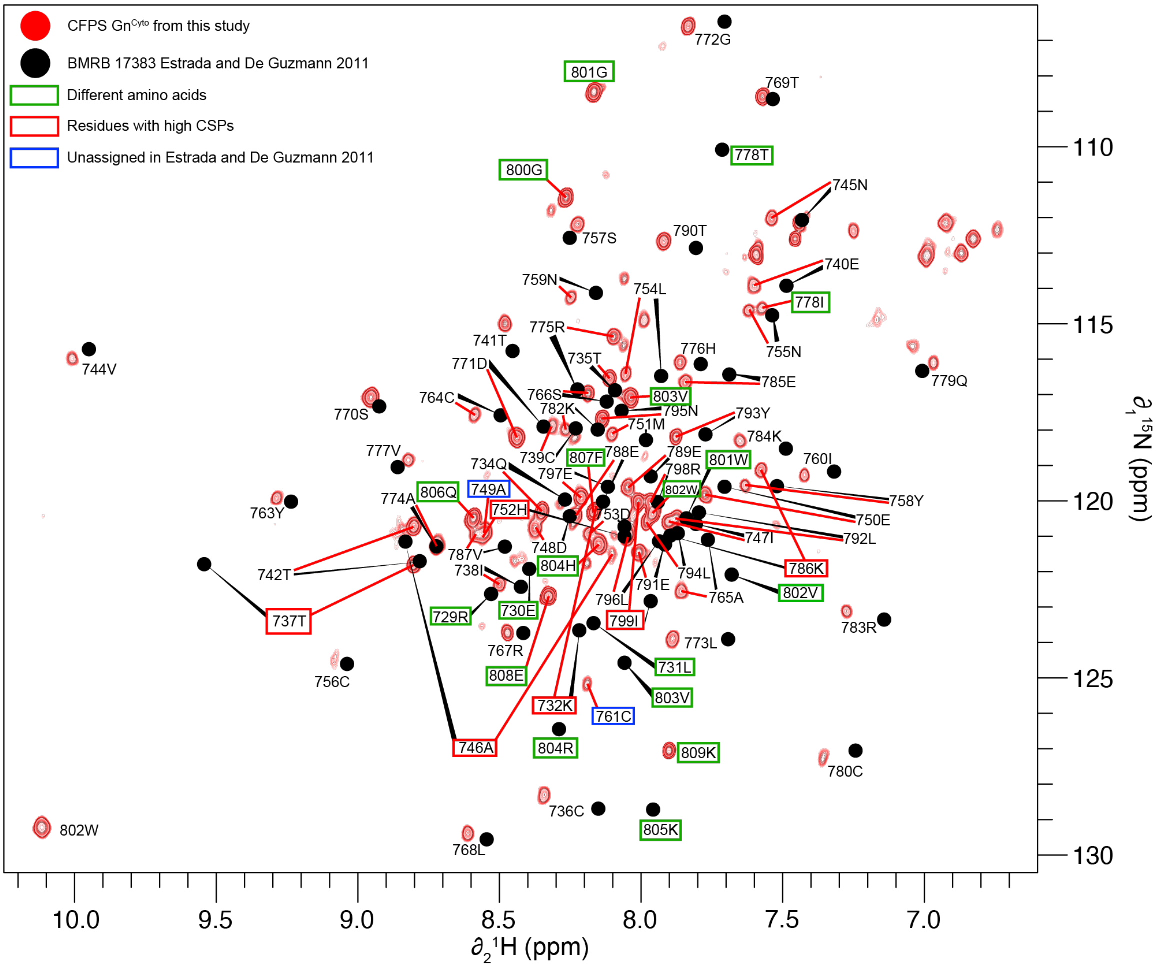


```
              730       740       750       760       770       780       790       800
IbAr10200   MKPQTCTICETTPVNAIDAEMHDLNCSYNICPYCASRLTSDGLARHVIQCPKRKEKVEETELYLNLERIGGWSHPQFEK
SPU103/87  RELKPQTCTICETTPVNAIDAEMHDLNCSYNICPYCASRLTSDGLARHVTQCPKRKEKVEETELYLNLERIPWVVRK
```

Figure S3: Comparison between the ^{15}N HSQC spectrum recorded on the CFPS ^{2}H-^{13}C-^{15}N Gn^{cyto} sample in red and a simulation using the chemical shifts deposited in the BMRB 17383 (Estrada and De Guzman, 2011). The comparison of the amino-acid sequences of Gn^{cyto} analyzed in this study (IbAr10200) and from (Estrada and De Guzman, 2011) (SPU103/87) is shown at the bottom. The amino acids which are different between the two sequences are highlighted in green.

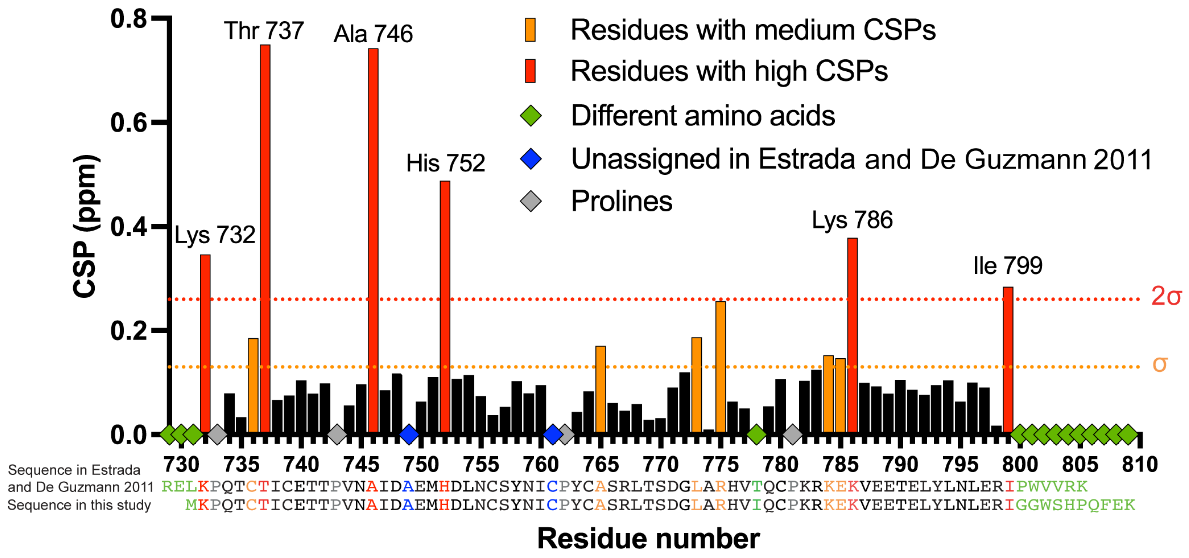

Figure S4: CSPs, calculated according to $CSP = \sqrt{(\partial^1 H_1 - \partial^1 H_2)^2 + \left(\frac{\gamma^{15}N}{\gamma^1 H}(\partial^{15}N_1 - \partial^{15}N_2)\right)^2}$, where $\partial^1 H$ stands for the amide proton chemical shift, $\partial^{15}N$ is the amide nitrogen chemical shift, $\gamma^{15}N$ is the gyromagnetic ratio of nitrogen and $\gamma^1 H$ the proton gyromagnetic ratio. Differences in the amino-acid sequence are highlighted.

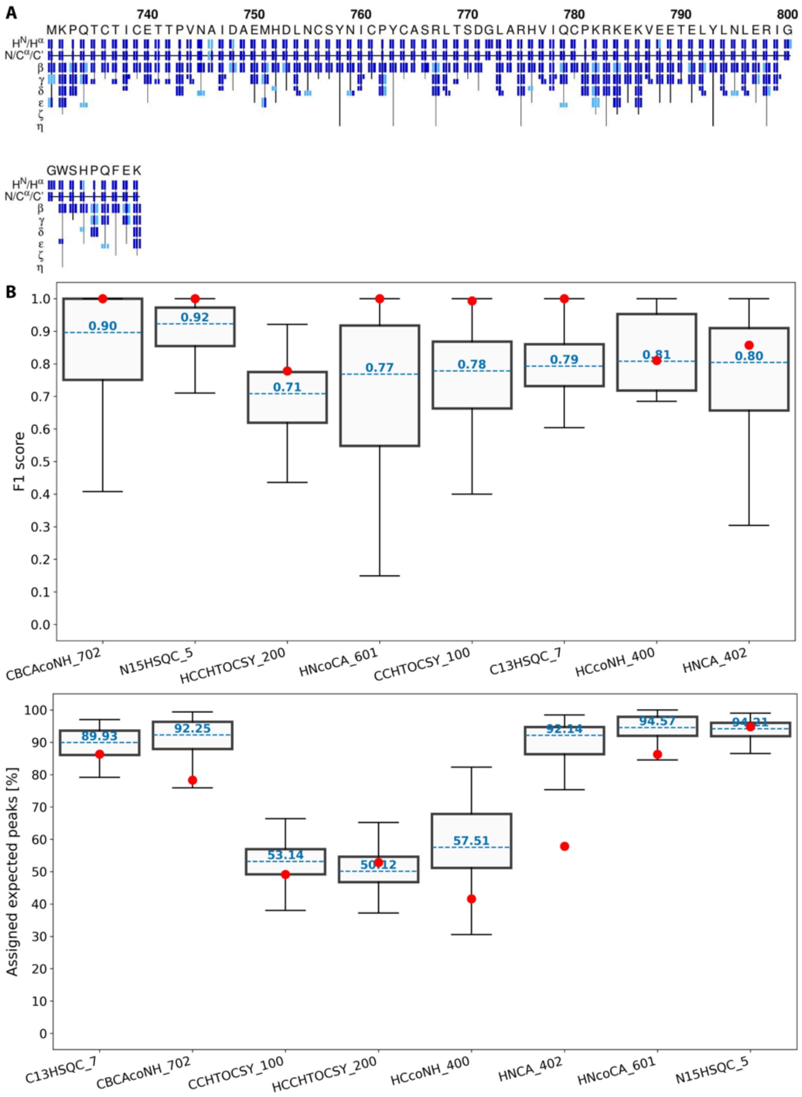

Figure S5: NMRtist output graph, F1 score and percentage of assigned expected peaks in the different spectra. (A) Extent and reliability of assignments obtained with the NMRtist automated resonance assignment algorithm using automatically picked peak lists. Each assignment for an atom is represented by a blue rectangle. Light colors indicate an assignment classified as weak by the chemical shift consolidation. (B) The top box plots show the distribution of the F1 scores of the spectra, and the red dots represent the F1 scores calculated for the output of the peak picking application call. Scores above the median (dotted line) indicate that the automated peak picking routine was able to process a spectrum more accurately than 50% of the spectra in the benchmark (https://nmrtist.org/static/public/examples/ARTINA/ARTINA_dataset3.html). The bottom plot shows the percentage of assigned peaks with respect to the expected number of cross peaks in each spectrum, as given by the protein sequence.

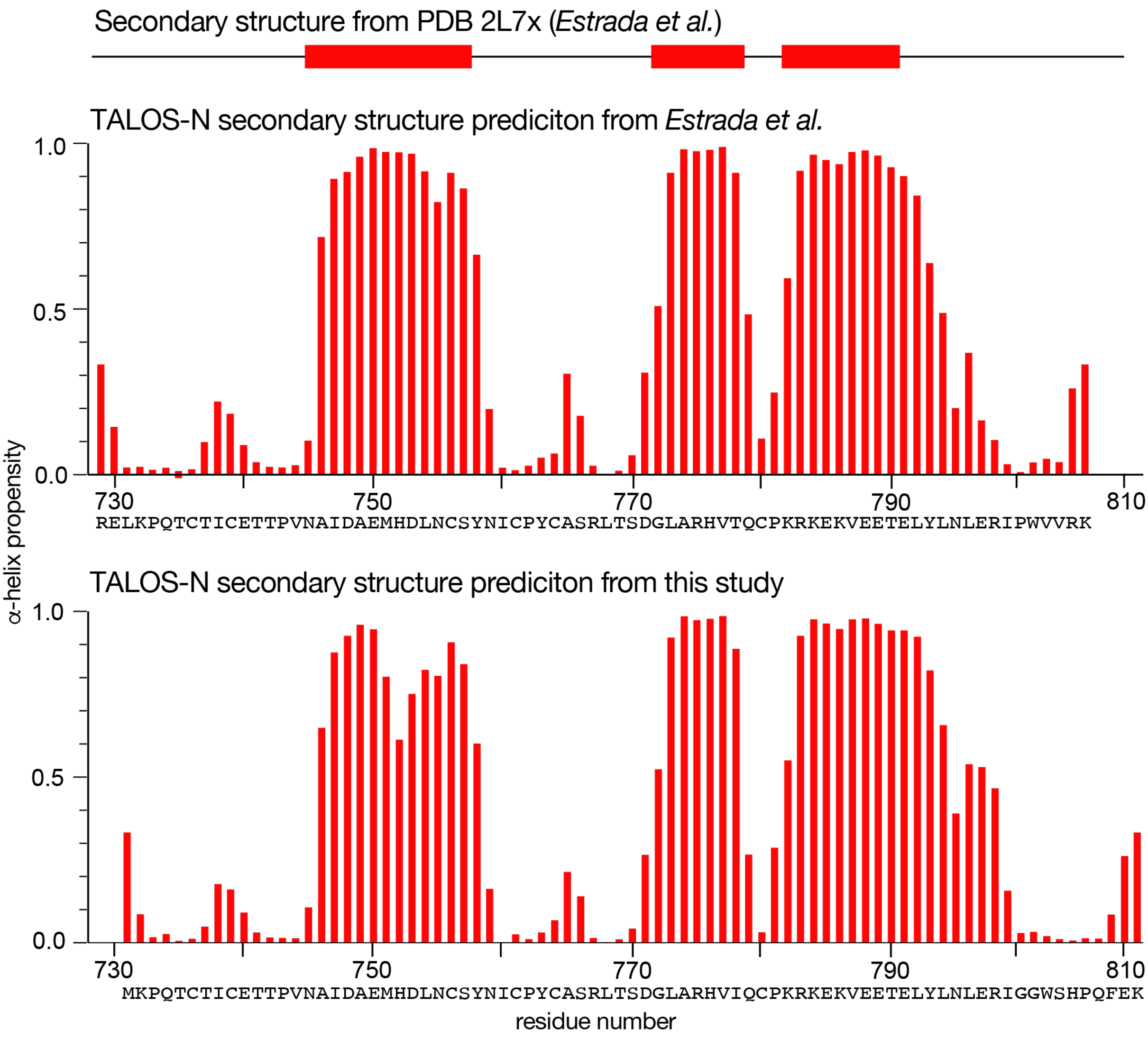

Figure S6: Comparison of the secondary structure predicted by TALOS-N from (Estrada and De Guzman, 2011) chemical shifts (top) and the chemical shifts from this study (bottom). The secondary structure elements match, confirming that the folding of Gn is maintained. The α-helices from the structure (PDB 2L7x) (Estrada and De Guzman, 2011) are shown in red on top.

Table S1: Spectra recorded and sample used

**^{2}H-^{13}C-^{15}N Gn^{cyto}**	**^{13}C-^{15}N Gn^{cyto}**
^{15}N-HSQC	^{15}N-HSQC
HNCA	^{13}C-HSQC
HNCO	HccoNH-TOCSY
HNcoCA	hCCH-TOCSY
HNcoCACB	HCcH-TOCSY

References

Estrada, D. F. and De Guzman, R. N.: Structural Characterization of the Crimean-Congo Hemorrhagic Fever Virus Gn Tail Provides Insight into Virus Assembly, Journal of Biological Chemistry, 286, 21678–21686, https://doi.org/10.1074/jbc.M110.216515, 2011.
